# Long non-coding RNA OIP5-AS1 aggravates acute lung injury by promoting inflammation and cell apoptosis via regulating the miR-26a-5p/TLR4 axis

**DOI:** 10.1186/s12890-021-01589-1

**Published:** 2021-07-14

**Authors:** Qingsong Sun, Man Luo, Zhiwei Gao, Xiang Han, Weiqin Wu, Hongmei Zhao

**Affiliations:** Department of Emergency, The Affiliated Huaian No.1 People’s Hospital of Nanjing Medical University, No. 1, Huanghe West Road, Huaiyin District, Huaian, 223300 Jiangsu China

**Keywords:** OIP5-AS1, miR-26a-5p, TLR4, Acute lung injury

## Abstract

**Background:**

Acute lung injury (ALI) is a pulmonary disorder that leads to acute respiration failure and thereby results in a high mortality worldwide. Increasing studies have indicated that toll-like receptor 4 (TLR4) is a promoter in ALI, and we aimed to explore the underlying upstream mechanism of TLR4 in ALI.

**Methods:**

We used lipopolysaccharide (LPS) to induce an acute inflammatory response in vitro model and a murine mouse model. A wide range of experiments including reverse transcription quantitative polymerase chain reaction, western blot, enzyme linked immunosorbent assay, flow cytometry, hematoxylin–eosin staining, RNA immunoprecipitation, luciferase activity and caspase-3 activity detection assays were conducted to figure out the expression status, specific role and potential upstream mechanism of TLR4 in ALI.

**Result:**

TLR4 expression was upregulated in ALI mice and LPS-treated primary bronchial/tracheal epithelial cells. Moreover, miR-26a-5p was confirmed to target TLR4 according to results of luciferase reporter assay. In addition, miR-26a-5p overexpression decreased the contents of proinflammatory factors and inhibited cell apoptosis, while upregulation of TLR4 reversed these effects of miR-26a-5p mimics, implying that miR-26a-5p alleviated ALI by regulating TLR4. Afterwards, OPA interacting protein 5 antisense RNA 1 (OIP5-AS1) was identified to bind with miR-26a-5p. Functionally, OIP5-AS1 upregulation promoted the inflammation and miR-26a-5p overexpression counteracted the influence of OIP5-AS1 upregulation on cell inflammatory response and apoptosis.

**Conclusion:**

OIP5-AS1 promotes ALI by regulating the miR-26a-5p/TLR4 axis in ALI mice and LPS-treated cells, which indicates a promising insight into diagnostics and therapeutics in ALI.

**Supplementary Information:**

The online version contains supplementary material available at 10.1186/s12890-021-01589-1.

## Introduction

Acute lung injury (ALI), a severe respiratory disorder, is characterized by heterogeneous pathologic factors [1, 2]. With a high morbidity and mortality, ALI has posed a huge threat to human life and health globally [3, 4]. Previous studies have verified that ALI was closely associated with acute inflammatory response [5–8], and large numbers of studies have attempted to find an effective therapy for ALI, while the mortality rates of ALI patients are sorrowfully high [4, 9]. Therefore, it is crucial to explore the potential molecular mechanisms underlying inflammatory response for the improvement of ALI clinical therapy.

Long noncoding RNAs (lncRNAs) are longer than 200 nucleotides and participate in many biological and physiological processes [10, 11]. It is widely accepted that lncRNAs act as miRNA “sponges” to compete with mRNAs for miRNAs with shared miRNAs responses elements and can regulate miRNAs [11–13].

Importantly, it is reported that this ceRNA regulatory network is broadly implicated in multiple diseases including ALI [14–16]. For instance, the knockdown of lncRNA X-inactive specific transcript mitigates primary graft dysfunction by sponging miR-21 and targeting IL-12A following lung transplantation [17]. Additionally, cancer susceptibility 2 was identified to inhibit lung epithelial cell apoptosis by targeting the miR-144-3p/aquaporin 1 axis, and thereby improve ALI [18]. Recently, lncRNA OPA-interacting protein 5 antisense transcript 1 (OIP5-AS1) has been reported to involve in pathogenesis of diverse diseases including tumors [19, 20], myocardial ischemia/reperfusion injury [21], and osteoarthritis [22]. Moreover, OIP5-AS1 was revealed to regulate cell injury and inflammatory response in atherosclerosis and rheumatoid arthritis [23, 24]. However, the role of OIP5-AS1 in ALI remains to be studied.

MicroRNAs (miRNAs), another group of non-coding RNAs, comprise about 22 nucleotides and can post-transcriptionally regulate gene expression [25, 26]. For example, miR-126 blocks the development of coronary atherosclerosis in mice via targeting sphingosine-1-phosphate receptor 2 [27]. In addition, miR-38 protects endothelial cell against inflammatory damage in coronary heart disease via targeting C-X-C motif chemokine receptor 4 [28]. Previously, miR-26a-5p expression was confirmed to be increased in synovial tissues of patients with rheumatoid arthritis and elevates the invasion ability of synovial fibroblasts via targeting Smad 1 [29]. MiR-26a-5p negatively modulates the development of neuropathic pain in CCI rat models via targeting mitogen-activated protein kinase 6 [30]. Nevertheless, the potential target mRNAs of miR-26a-5p in ALI remain to be elucidated.

Accumulating studies have revealed that toll like receptor 4 (TLR4) is a key regulator of inflammatory response [31, 32]. For example, TLR4 aggravates the inflammation and apoptosis of retinal ganglion cells in high glucose [33]. In addition, TLR4 silence decreases the inflammation, which further prevents the kidney damage and the development of fibrosis in cyclosporine nephrotoxicity [34]. However, the role of TLR4 in ALI deserves a further exploration.

In this study, we established animal and cell models of ALI by LPS treatment to explore the role as well as the regulatory function of OIP5-AS1 in ALI. We found out that OIP5-AS1 aggravated ALI development by promoting inflammation via regulating the miR-26a-5p/TLR4 axis. Our findings might offer a promising approach for ALI treatment.

## Materials and methods

### ALI mice model

Total 64 BALB/c mice were raised in a room at 25 °C in a light/dark cycle of 12 h/12 h. All the mice were divided into two groups randomly: sham group (n = 8) and ALI group (n = 54). For establishment of the ALI mice model, 10 μg of *Escherichia coli* O111:B4-derived lipopolysaccharide (LPS; Sigma‐Aldrich Inc., USA) in 50 μL of phosphate buffer saline (Sigma‐Aldrich) was intratracheally instilled while the mice in the sham group were injected with an equivalent volume of PBS (Sigma‐Aldrich). After 6 h, the mice were anesthetized with 1.5% pentobarbital sodium (60 mg/kg) and then sacrificed by cervical dislocation under anesthesia, and lung tissues were collected for following biochemical assays. Macrophages and neutrophils were isolated from ALI mice and sham mice according to previous studies [35, 36]. All experimental procedures were based on the National Institutes of Health Guidelines for the Care and Use of Laboratory Animals and approved by the Ethics Committee of the Affiliated Huaian No.1 People's Hospital of Nanjing Medical University. All methods were conducted in accordance with the ARRIVE guidelines (https://arriveguidelines.org).

### Wet/dry ratio of the lungs

The examination of lung W/D weight ratio was used to evaluate pulmonary edema. After 6 h of LPS treatment, the mice were euthanized, and the fresh lung tissues were weighed and the weight was recorded, following the drying at 180 °C in an oven for 24 h to examine dry weight.

### Histological analysis

The mouse lung tissue samples were immobilized with 10% formalin for one day. After embedding in the paraffin, the tissues were cut into sections at the thickness of 5 μm. Furthermore, the tissue pieces were stained with hematoxylin–eosin (H&E) solution at room temperature and observed with a light microscope (Nikon, Japan). The following standards were used to score the lung injury, 0: no damage or minimal damage; 1: mild damage; 2: moderate damage; 3: severe damage; 4: diffuse injury.

### Adeno-associated virus injection

Mice received an intratracheal injection of an adeno-associated virus 6 (AAV6) system as described previously [37]. AAVs carrying miR-26a-5p, TLR4, OIP5-AS1, and the empty vectors were synthesized by Hanheng Biotechnology Co., Ltd. (Shanghai, China).

### Cell culture and transfection

The human embryonic lung fibroblast WI-38, mice lung epithelial TC-1 cells (Cell Bank of Type Culture Collection of Chinese Academy of Sciences, Shanghai, China), human primary bronchial/tracheal epithelial cells (PBECs; catalogue cumber: PCS-300-010; ATCC) were maintained in Dulbecco's modified Eagle medium (Sigma-Aldrich) containing 5% fetal bovine serum (Gibco, USA), 100 U/mL penicillin and 100 μg/mL streptomycin at 37 °C in a 5% CO_2_ atmosphere. For cell transfection, 50 nM miR-26a-5p mimics (5′-UUCAAGUAAUCCAGGAUAGGCU-3′), 50 nM anti-miR-26a-5p (5′-AGCCUAUCGAUAUACUUGAA-3′), 1 μg of pcDNA3.1/OIP5-AS1, 1 μg of pcDNA3.1/TLR4 with corresponding negative controls were transfected into WI-38, TC-1 cells and PBECs using 3 μL of Lipofectamine 2000 (Invitrogen, USA). After 48 h of transfection, the cells were treated with 100 ng/mL LPS for 6 h and harvested for further use. Vectors were obtained from Genepharma (Shanghai, China).

### Reverse transcription quantitative polymerase chain reaction (RT-qPCR)

Total RNA was extracted from cells or tissues with TRIzol reagent (Invitrogen; Thermo Fisher Scientific, Inc. USA). For miRNA analysis, the extracted miRNA (1 µg) was reverse transcribed into complementary DNA with a TaqMan MicroRNA Reverse Transcription Kit (Invitrogen). For lncRNA and mRNA analysis, RNA (1 µg) was reverse transcribed into complementary DNAs using the Oligo dT primer (Invitrogen). PCR reactions were conducted on an ABI 7500 Real-Time PCR System (Applied Biosciences, USA) with the following PCR procedure: initial denaturation at 95 °C for 30 s, with 40 cycles of denaturation at 95 °C for 5 s, as well as annealing at 60 °C for 20–30 s. The gene levels were determined by the 2^−ΔΔCt^ method [38] and were normalized to GAPDH or U6 expression levels. The primers used for RT-qPCR were as follows:

- hOIP5-AS1
- qPCR: F: 5′-AACAGGTGCTTAGGTGGTGG-3′,
  - R: 5′-TGGCACTGCATGAGGGATTT-3′;
- mOIP5-AS1
- qPCR: F: 5′-AAGCACAGTTGACCGCAGTA-3′,
  - R: 5′-CCAACCCAGTCTCACATGCT-3′;
- hBax
- qPCR: F: 5′-TCATGGGCTGGACATTGGAC-3′,
  - R: 5′-GCGTCCCAAAGTAGGAGAGG-3′;
- mBax
- qPCR: F: 5′-CTGGATCCAAGACCAGGGTG-3′,
  - R: 5′-CTTCCAGATGGTGAGCGAGG-3′;
- hBcl-2
- qPCR: F: 5′-TTTGAGTTCGGTGGGGTCAT-3′,
  - R: 5′-AGAAATCAAACAGAGGCCGCA-3′;
- mBcl-2
- qPCR: F: 5′-AACATCGCCCTGTGGATGAC-3′,
  - R: 5′-TGCACCCAGAGTGATGCAG-3′;
- hIL-1β
- qPCR: F: 5′-TGAGCTCGCCAGTGAAATGA-3′,
  - R: 5′-CATGGCCACAACAACTGACG-3′;
- mIL-1β
- qPCR: F: 5′-TGCCACCTTTTGACAGTGATG-3′,
  - R: 5′-TGATGTGCTGCTGCGAGATT-3′;
- hTNF-α
- qPCR: F: 5′-CTGGGGCCTACAGCTTTGAT-3′,
  - R: 5′-GGCCTAAGGTCCACTTGTGT-3′;
- mTNF-α
- qPCR: F: 5′-ACTGAACTTCGGGGTGATCG-3′,
  - R: 5′-GTTTGCTACGACGTGGGCTA-3′;
- hGAPDH
- qPCR: F: 5′-GCTCTCTGCTCCTCCTGTTC-3′,
  - R: 5′-GACTCCGACCTTCACCTTCC-3′;
- mGAPDH
- qPCR: F: 5′-GGAGAGTGTTTCCTCGTCCC-3′,
  - R: 5′-ATGAAGGGGTCGTTGATGGC-3′;
- hTLR4
- qPCR: F: 5′-GACGGTGATAGCGAGCCAC-3′,
  - R: 5′-TTAGGAACCACCTCCACGCAG-3′;
- mTLR4
- qPCR: F: 5′-CCTGTGGACAAGGTCAGCAA-3′,
  - R: 5′-CTCGGCACTTAGCACTGTCA-3′;
- hsa-miR-26a-5p
- qPCR: F: 5′-GCGCGCGTAACAGTCTACAGC-3′,
  - R: 5′-GTCGTATCCAGTGCAGGGTCC-3′;
- mmu-miR-26a-5p
- qPCR: F: 5′-TCGGCAGGTTCAAGTAATCC-3′,
- R: 5′-CTCAACTGGTGTCGTGGAGT-3′;U6 (human)
- qPCR: F: 5′-CTCGCTTCGGCAGCACA-3′,
  - R: 5′-AACGCTTCACGAATTTGCGT-3′;
- U6 (mouse)
- qPCR: F: 5′-CGCACTTTACGGCTACCTCT-3′,
  - R: 5′-GCGACAAGGGAAGGGAACAA-3′;

### RNA immunoprecipitation (RIP) assay

The RIP assay was conducted using a Magna RIP RNA-Binding Protein Immunoprecipitation Kit (Millipore, USA). Briefly, cell lysate was centrifuged at 12,000×*g* for 30 min and the supernatant were collected. Ago2 antibody (1:500; Otwo Biotech, Shenzhen, China) or IgG (1:100; Sigma, USA) were respectively incubated with 20 μL of protein G-agarose beads for 2 h at 4 °C and then cell lysate supernatant was filled in and incubated overnight at 4 °C. RNA was isolated from magnetic beads with TRIzol reagent (Invitrogen) and RT-qPCR was used to detect OIP5-AS1 and miR-26a-5p expression levels in the precipitates.

### Western blot analysis

Lung tissues and cells were harvested and lysed by protein lysis buffer (Bio-Rad Laboratories). Then equal amount of protein samples (50 μg/lane) was separated on 12% sodium dodecyl sulfate polyacrylamide gel electrophoresis followed by transferring onto polyvinylidene difluoride membranes. Blocked by 5% skimmed milk for 1 h at room temperature, the primary antibodies against TLR4 (ab13556; 1/500; Abcam, UK), Bax (ab182733; 1/2000; Abcam), Bcl-2 (ab 182,858; 1/2000; Abcam) and GAPDH (ab181602; 1/10000; Abcam) were incubated at 4 °C overnight. After washing by Tris-Buffered Saline (Bio-Rad Laboratories), the membranes were then cultured with horseradish peroxidase-conjugated secondary antibodies (1/10000; Abcam) at room temperature for 2 h. At last, the protein bands were assessed via an ECL kit (Amersham Biosciences) and the intensity was analyzed using ImageJ software. Original western blotting gels were provided in Additional file 1.

### Luciferase reporter assay

The luciferase reporter vectors (pGLO) containing the wild type 3′ untranslated region (3′ UTR) of TLR4 complementary to miR-26a-5p or mutated 3′ UTR of TLR4 were constructed by Genepharma (Shanghai, China), and were termed as pGLO-TLR4 3′ UTR Wt or pGLO-TLR4 3′ UTR Mut. The pGLO-miR-26a-5p -Wt or pGLO-miR-26a-5p-Mut was also constructed. Afterwards, the vectors (1 µg) were cotransfected with 50 nM miR-26a-5p, 50 nM anti-miR-26a-5p into PBECs, TC-1 and WI-38 cells using Lipofectamine 2000 (Invitrogen), separately. After 48 h, the relative luciferase activities were detected with luciferase reporter assay system (Promega, USA). Relative luciferase activity was calculated as the ratio of firefly luciferase activity to Renilla luciferase activity.

### Flow cytometry assay

The apoptosis rate of PBECs, TC-1 and WI-38 cells was evaluated using an Annexin V fluorescein isothiocyanate/propidium iodide (Annexin V-FITC/PI) apoptosis assay kit (Invitrogen) following previous procedures [39]. In brief, PBECs, transfected TC-1 and WI-38 cells were resuspended to a concentration of 4 × 10^5^ cells/mL and subjected to staining with 5 μL of Annexin V-FITC and PI for 25 min in the dark. Afterwards, the flow cytometry (FACS 420, BD Biosciences, USA) was used to analyze apoptotic cells, and the data was analyzed using BD CellQuest Pro version 1.2 software (BD Biosciences). Percentage of apoptosis rate was calculated as apoptotic cells/total cells × 100%.

### The caspase-3 activity detection

The caspase-3 activity in lung tissues and cells was measured by a caspase-3 activity kit (Beyotime) according to the manufacture’s protocols. The tissues were isolated, prepared and lysed in cell lysis buffer. After protein concentration analyzing, proteins were filled in the cell lysis buffer. Subsequently, the reaction buffer and DEVD-ρNA substrate (caspase-3) were supplemented into the lysis buffer. The reaction mixtures were cultured at 37 °C for 2 h. The absorbance at 405 nm was assessed via a microplate reader (Meigu, Shanghai, China).

### Enzyme linked immunosorbent assay (ELISA)

ELISA was performed using the ELISA kits for TNF-α and IL-1β (ab181421 and ab214025; Abcam, USA) in order to detect the concentrations of TNF-α and IL-1β in culture supernatant of PBECs after centrifugation 12,000×*g* for 10 min at 4 °C. Absorbance at 450 nm was determined via a microplate reader according to manufacturer’s protocols.

### Statistical analysis

Data were presented as means ± standard deviation, as shown by the histograms with error bars in the figures. All data were assessed for normal distribution (Shapiro–Wilk test) and homogeneity of variance (Bartlett’s test). The differences between 2 groups were evaluated by independent Student’s tests and that among 3 or more groups were evaluated by one-way analysis of variance followed by Tukey’s post hoc test. *P* value less than 0.05 was considered significant. All experiments were repeated at least three times.

## Results

### TLR4 is upregulated in ALI mice and LPS-treated cells

**Fig. 1:**
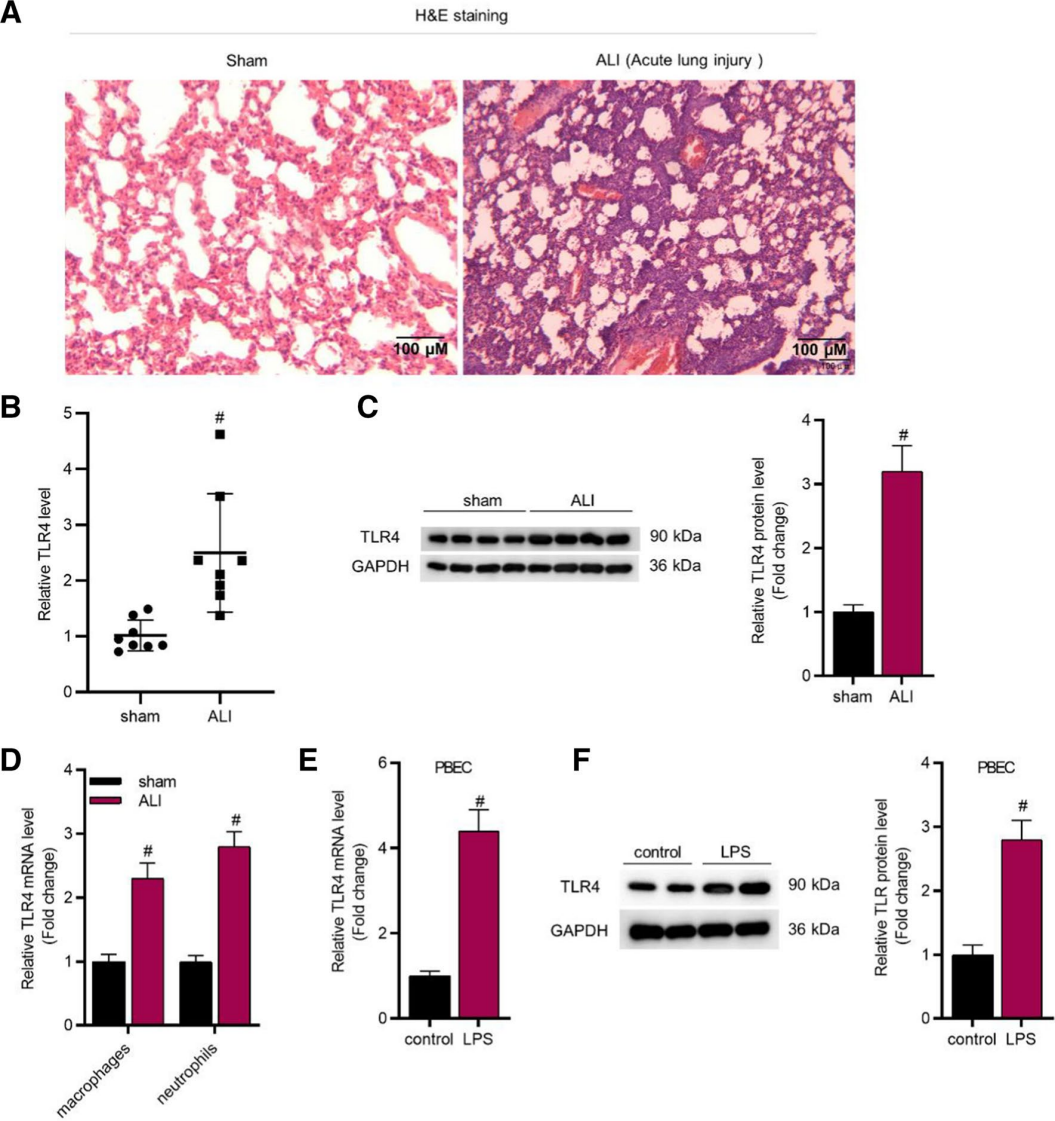
TLR4 was upregulated in ALI mice and LPS-stimulated cells. **A** H&E staining was conducted to assess the lung injury degree in ALI mice (magnification: × 100). **B**, **C** The mRNA and protein expression of TLR4 in mice’s lung tissues (n = 8 per group) was detected by RT-qPCR and western blot analyses. **D**, Relative TLR4 expression in lung resident macrophages and neutrophils isolated from sham or ALI mice. **E**, **F** The mRNA and protein expression of TLR4 in LPS-stimulated PBECs was detected by RT-qPCR and western blot analyses. ^#^*P* < 0.05 compared with Sham group in **B**–**D**; ^#^*P* < 0.05 compared with control group in **E**, **F**

To investigate the potential role of TLR4 in lung injury, the ALI mice model was established via intratracheally instilling with LPS. The intact alveolar structure without thickening or lymphocyte infiltration was observed in the lungs of sham-operated mice (left panel, Fig. 1A). Pulmonary lesions were found in ALI mice, and pathologically thickened alveolar walls, collapsed alveoli and infiltrated inflammatory cells were also observed in ALI mice (right panel, Fig. 1A), suggesting that our ALI mice model was successfully established. Further, RT-qPCR and western blot were conducted to respectively investigate the mRNA and protein expression of TLR4. The data revealed that TLR4 was significantly upregulated in lung tissues of ALI mice (Fig. 1B, C). Figure 1D revealed that TLR4 expression was higher in macrophages and neutrophils isolated from ALI mice than sham mice. Figure 1E, F revealed that TLR4 expression was upregulated by LPS stimulation in PBECs. In addition, as revealed in Additional file 2: Fig. S1A, TLR4 expression was upregulated by LPS stimulation in WI-38 cells.

### TLR4 is upregulated by LPS stimulation and negatively modulated by miR-26a-5p

**Fig. 2:**
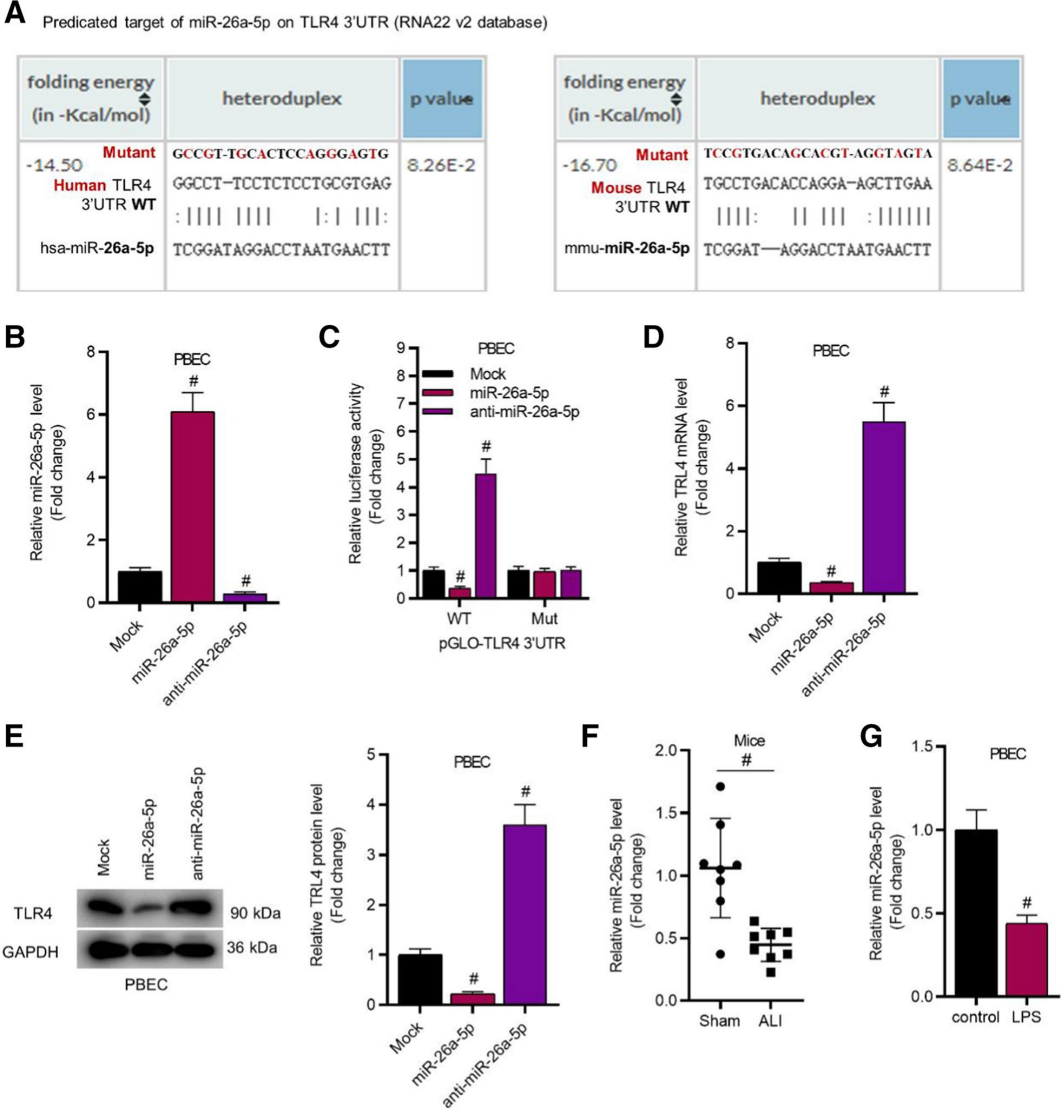
TLR4 was upregulated by LPS stimulation and negatively modulated by miR-26a-5p in PBECs. **A** RNA22 v2 database predicted the binding sequences of miR-26a-5p and TLR4. **B** RT-qPCR was conducted to assess the efficiency of miR-26a-5p overexpression and miR-26a-5p knockdown in PBECs. **C** Luciferase reporter assay was performed to verify the interaction between miR-26a-5p and TLR4 in PBECs. **D**, **E** RT-qPCR and western blot assay were applied for detecting the effects of miR-26a-5p overexpression and miR-26a-5p knockdown on the mRNA and protein expression of TLR4 in PBECs. **F**, **G** The expression of miR-26a-5p in mice’s lung tissues (n = 8 per group) and LPS-treated PBECs was assessed by RT-qPCR analysis. ^#^*P* < 0.05 compared with Mock group in **B**–**E**; ^#^*P* < 0.05 compared with Sham group in **F**; ^#^*P* < 0.05 compared with control group in **G**

To find out the potential miRNAs that might bind with TLR4, we searched RNA22 v2 online website and found the underlying binding sites between miR-26a-5p and TLR4 (Fig. 2A). Next, miR-26a-5p expression was elevated by transfection with miR-26a-5p mimics and reduced via transfection with anti-miR-26a-5p in PBECs, as evidenced by the RT-qPCR analysis (Fig. 2B). For verifying the relationship between miR-26a-5p and TLR4, luciferase assay was then performed. The results disclosed that the luciferase activity of pmirGLO-TLR4-WT was decreased by miR-26a-5p mimics and increased by anti-miR-26a-5p, while the luciferase activity of pmirGLO-TLR4-Mut had no significant changes in response to miR-26a-5p mimics or anti-miR-26a-5p (Fig. 2C). These findings suggested that miR-26a-5p can bind with TLR4. Moreover, RT-qPCR and western blot analyses demonstrated that TLR4 expression at the mRNA and protein levels was decreased in the miR-26a-5p mimics group and increased in anti-miR-26a-5p group (Fig. 2D, E). Furthermore, miR-26a-5p level was significantly decreased in ALI mice and LPS-treated PBECs (Fig. 2F, G). Additional file 2: Fig. S1B–F revealed that miR-26a-5p negatively regulated TLR4 expression in WI-38 and TC-1 cells. Additional file 2: Fig. S1G revealed that miR-26a-5p level was decreased in LPS-treated WI-38 cells.

### *MiR-26a-5p overexpression suppresses the productions of inflammatory factors *via* targeting TLR4*

**Fig. 3:**
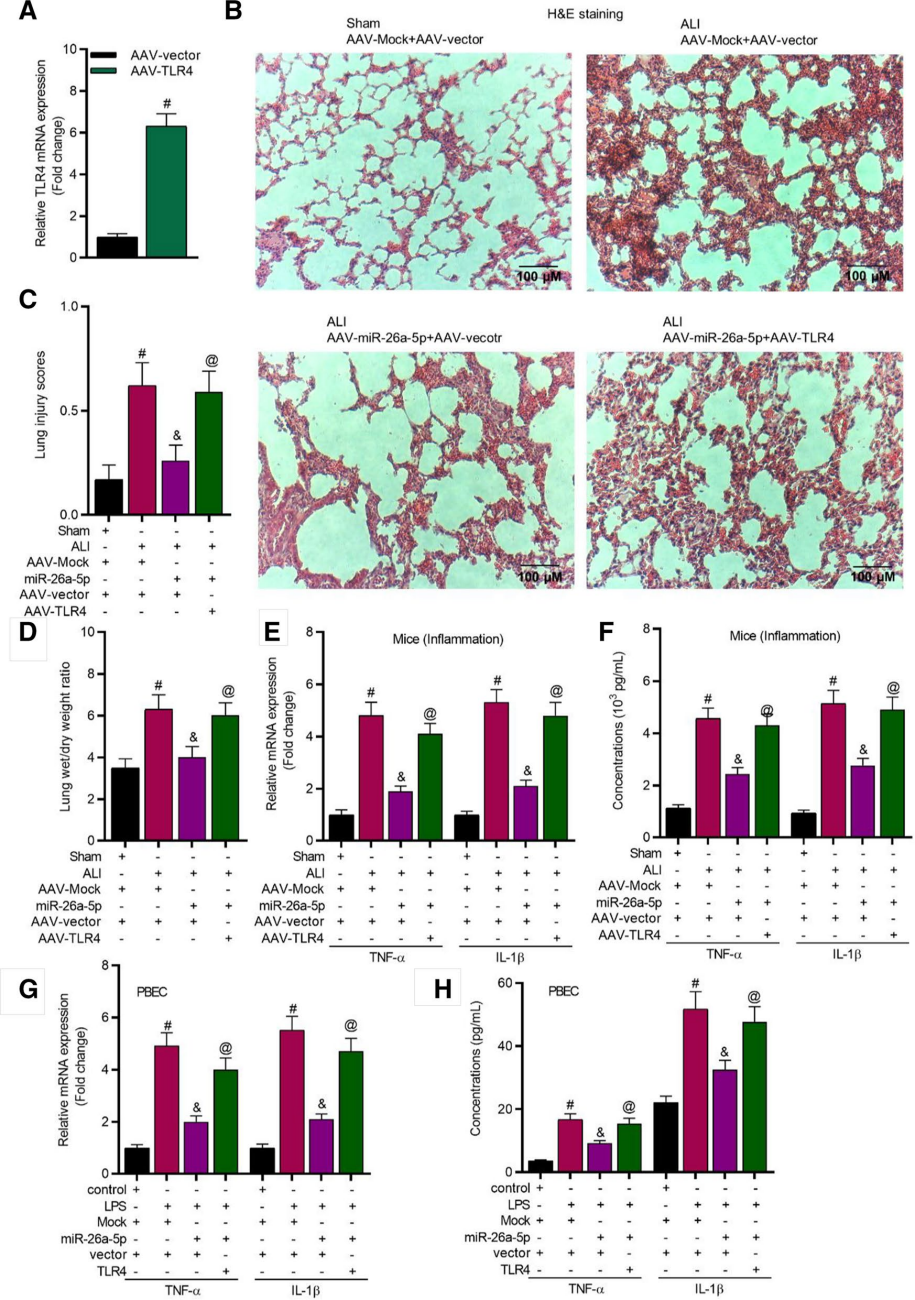
MiR-26a-5p inhibited the progression of lung injury by regulating TLR4. **A** TLR4 expression in lung tissues of mice (n = 8 per group) was evaluated by RT-qPCR. **B**, **C** H&E staining (magnification: × 100) was conducted to assess the lung injury degree (n = 8 per group). **D** Statistical analysis revealed the lung wet/dry weight ratio (n = 8 per group). **E**, **F** RT-qPCR and ELISA were performed to evaluate the levels of TNF-α and IL-1β in lung tissues of mice (n = 8 per group). **G**, **H** RT-qPCR and ELISA were performed to evaluate the levels of TNF-α and IL-1β in PBECs. ^#^*P* < 0.05 compared with AAV-vector group in A; ^#^*P* < 0.05 compared with Sham + AAV-Mock + AAV-vector group, ^&^*P* < 0.05 compared with ALI + AAV-Mock + AAV-vector group, ^@^*P* < 0.05 compared with ALI + AAV-miR-26a-5p + AAV-vector group in **C**–**F**; ^#^*P* < 0.05 compared with control + Mock + vector group, ^&^*P* < 0.05 compared with LPS + Mock + vector group, ^@^*P* < 0.05 compared with LPS + miR-26a-5p + vector group in **G**, **H**

Then, we explored whether miR-26a-5p influences lung inflammation by targeting TLR4. To begin with, TLR4 expression was effectively increased by AAV-TLR4 injection (Fig. 3A). Results of H&E staining assay revealed that miR-26a-5p overexpression alleviated the lung injury in ALI mice, while this effect was reversed by TLR4 upregulation (Fig. 3B, C). Moreover, we identified that miR-26a-5p overexpression reduced the lung wet/drying ratio, and upregulation of TLR4 rescued the effects (Fig. 3D). Furthermore, miR-26a-5p overexpression downregulated the contents of TNF-α and IL-1β in serum of ALI mice (Fig. 3E, F), and in LPS-stimulated PBEC supernatants (Fig. 3G, H), while TLR4 overexpression counteracted the effects. Additional file 2: Fig. S2A, B revealed that miR-26a-5p decreased TNF-α and IL-1β expression by targeting TLR4 in WI-38 and TC-1 cells.

### MiR-26a-5p overexpression reduces cell apoptosis by targeting TRL4

**Fig. 4:**
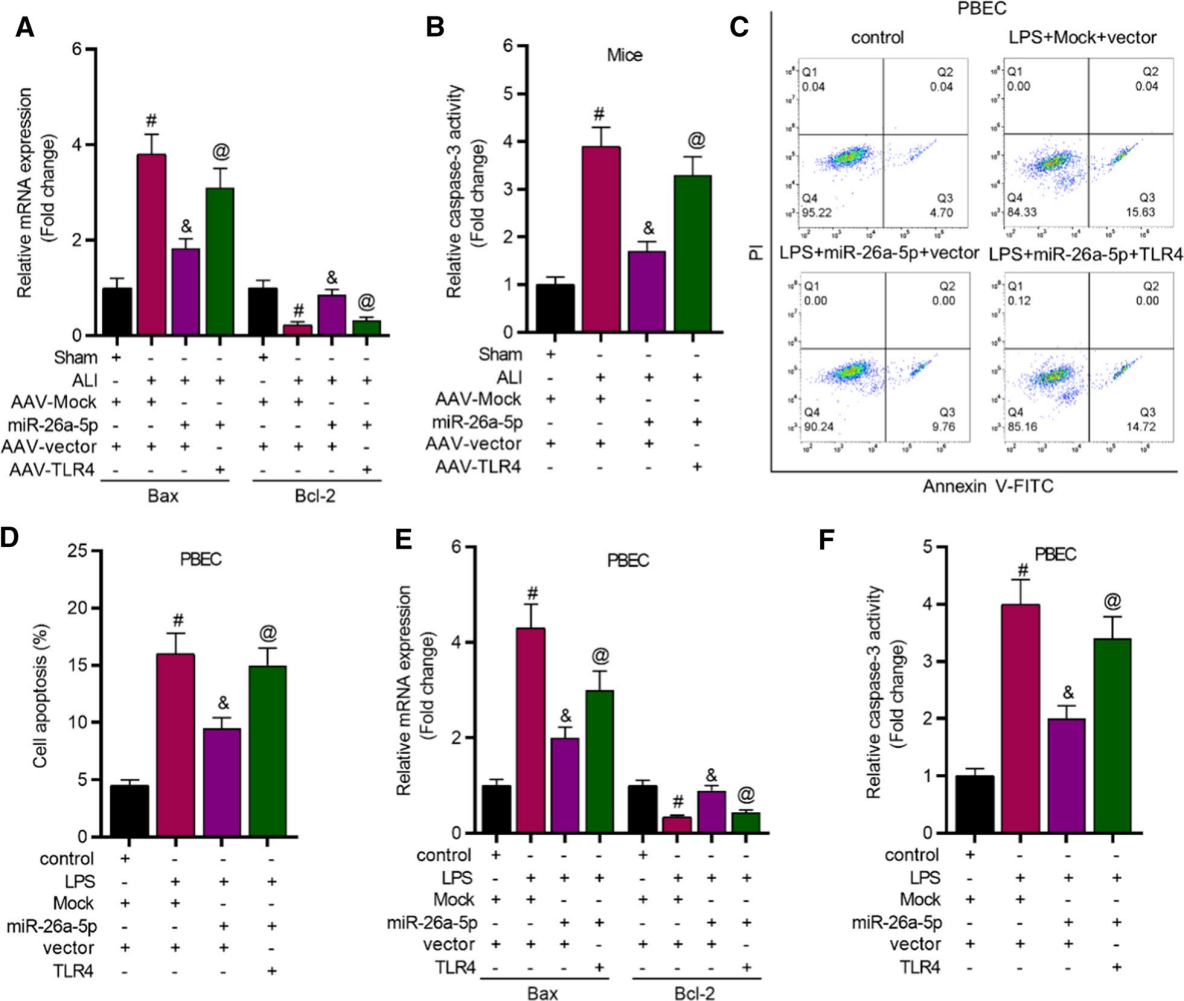
MiR-26a-5p overexpression alleviated cell apoptosis by modulating TLR4. **A** RT-qPCR was conducted to detect Bax and Bcl-2 expression in lung tissues of mice (n = 8 per group). **B** The activity of caspase-3 in mice’s lung tissues (n = 8 per group) was determined. **C** Flow cytometry assay was utilized to reveal cell apoptosis rate. **D** Bax and Bcl-2 expression in PBECs was calculated by RT-qPCR. **E** The activity of caspase-3 in PBECs was detected. ^#^*P* < 0.05 compared with Sham + AAV-Mock + AAV-vector group, ^&^*P* < 0.05 compared with ALI + AAV-Mock + AAV-vector group, ^@^*P* < 0.05 compared with ALI + AAV-miR-26a-5p + AAV-vector group in A, B; ^#^*P* < 0.05 compared with control + Mock + vector group, ^&^*P* < 0.05 compared with LPS + Mock + vector group, ^@^*P* < 0.05 compared with LPS + miR-26a-5p + vector group in **D**–**F**

MiR-26a-5p overexpression downregulated Bax expression and increased Bcl-2 expression in ALI mice while TLR4 upregulation reversed the effects (Fig. 4A). Moreover, the decreased activity of caspase-3 in ALI mice caused by elevation of miR-26a-5p was reversed by TLR4 overexpression (Fig. 4B). Furthermore, miR-26a-5p overexpression reduced cell apoptosis and TLR4 upregulation counteracted the effect of miR-26a-5p in cell apoptosis (Fig. 4C, D). In addition, the level of Bax was decreased while the level of Bcl-2 was upregulated by overexpression of miR-26a-5p in LPS-treated PBECs, while TLR4 upregulation inversely changed these effects (Fig. 4E). As presented in Fig. 4F, miR-26a-5p overexpression decreased the caspase-3 activity in PBECs, and TLR4 upregulation rescued the effects. Additional file 2: Fig. S2C–E revealed that miR-26a-5p inhibited apoptosis of WI-38 and TC-1 cells by targeting TLR4.

### OIP5-AS1 binds with miR-26a-5p

**Fig. 5:**
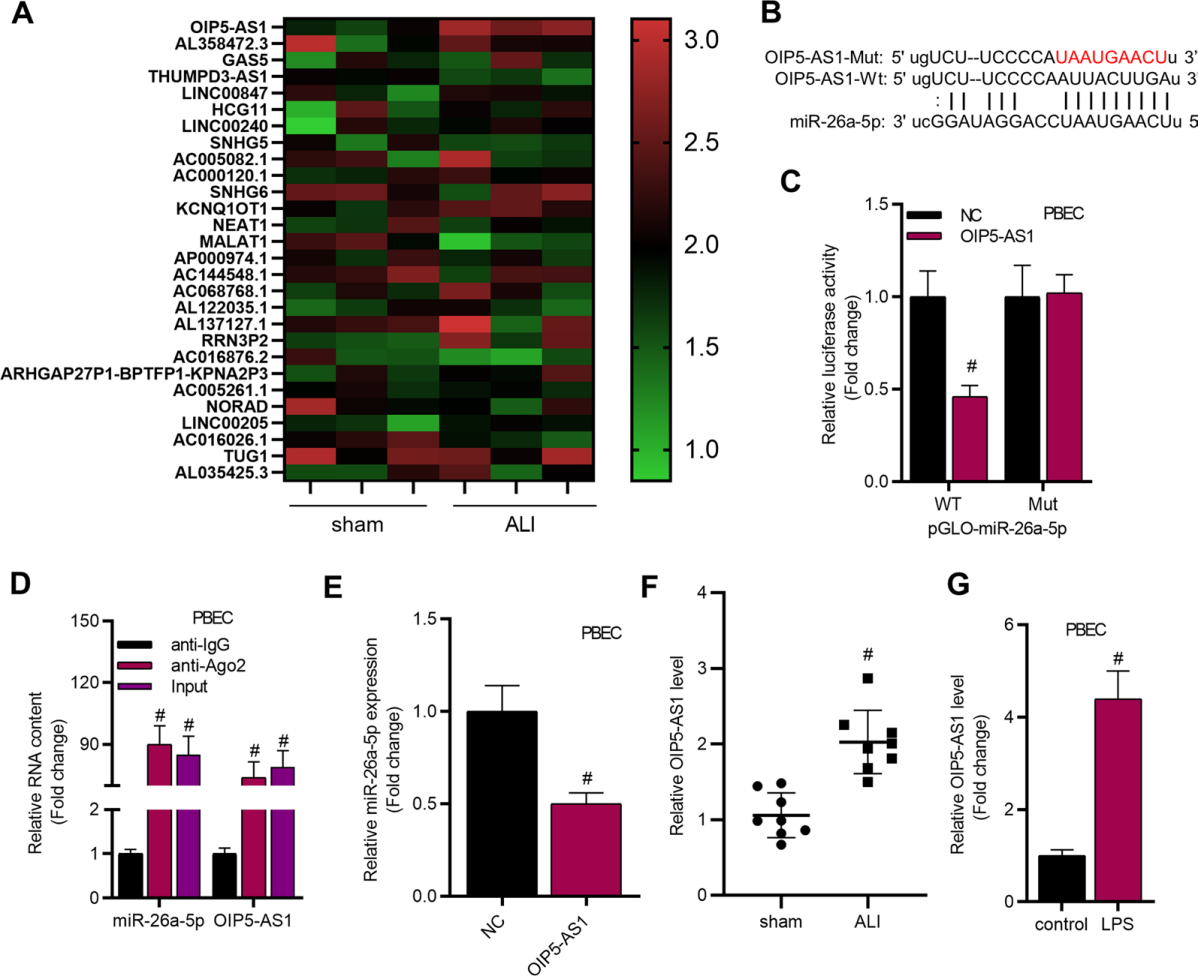
OIP5-AS1 regulated miR-26a-5p in ALI. **A** The expression of predicted lncRNAs in lung tissues from sham and ALI groups. **B** The predicted biding sites of miR-26a-5p on OIP5-AS1. **C**, **D** The interaction between miR-26a-5p and OIP5-AS1 was demonstrated by luciferase reporter and RIP assays. ^#^*P* < 0.05 compared with NC group or anti-IgG group. **E** The effects of overexpressed OIP5-AS1 on miR-26a-5p expression were estimated by RT-qPCR. ^#^*P* < 0.05 compared with NC group. **F**, **G** RT-qPCR analysis was conducted to evaluate the level of OIP5-AS1 in lung tissues of ALI mice and sham mice (n = 8 per group) and in LPS-stimulated PBECs. ^#^*P* < 0.05 compared with sham group or control group

According to starBase website (http://starbase.sysu.edu.cn), total 28 lncRNAs were identified for further exploration (condition: high stringency of CLIP Data; Additional file 2: Table S1). Expression of these lncRNAs was determined by RT-qPCR and shown in heat map in Fig. 5A. The heatmap displayed that only OIP5-AS1 was upregulated in all lung tissues from ALI group. We used starBase website and found the potential binding sites between OIP5-AS1 and miR-26a-5p (Fig. 5B). To validate the relationship between OIP5-AS1 and miR-26a-5p, luciferase reporter and RIP assay were conducted. Luciferase reporter assay showed that luciferase activity of pmirGLO-miR-26a-5p-WT was significantly decreased in OIP5-AS1 transfected cells, while no significant change was detected in pGLO-miR-26a-5p-Mut group (Fig. 5C). RIP assay indicated that OIP5-AS1 and miR-26a-5p were enriched in Ago2 groups but not in IgG groups (Fig. 5D). All these results illustrated that OIP5-AS1 can bind with miR-26a-5p. Then, we identified that OIP5-AS1 negatively regulated miR-26a-5p expression in PBECs (Fig. 5E). Thereafter, the data from RT-qPCR revealed that OIP5-AS1 was upregulated in ALI mice and LPS stimulated PBECs (Fig. 5F, G). Additional file 2: Fig. S3A–C revealed that OIP5-AS1 bound with miR-26a-5p and negatively regulated its expression in WI-38 and TC-1 cells. Additional file 2: Fig. S3D revealed that OIP5-AS1 was upregulated in LPS stimulated WI-38 cells.

### OIP5-AS1 increases the productions of inflammatory factors by sponging miR-26a-5p

**Fig. 6:**
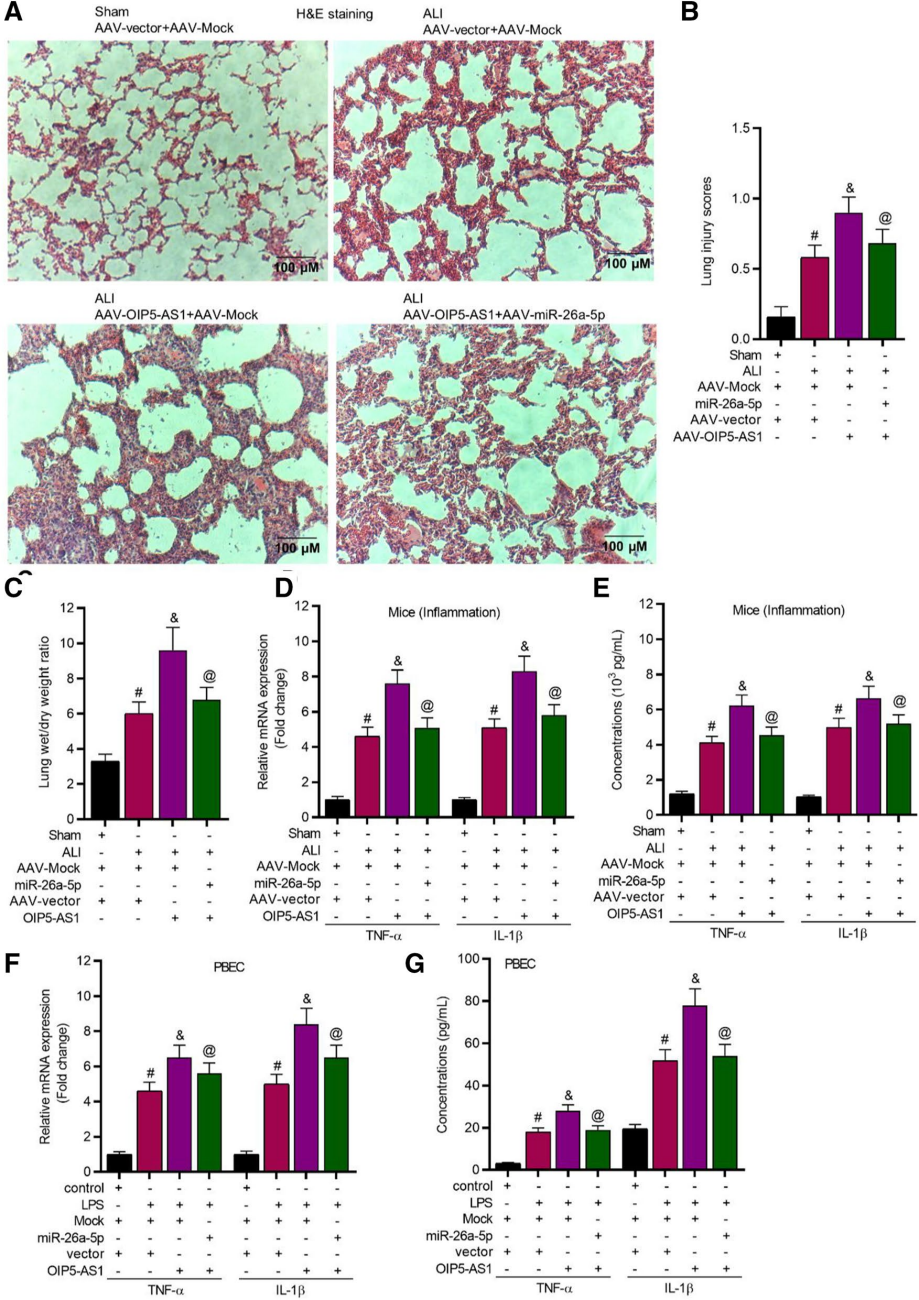
OIP5-AS1 promoted lung inflammation in ALI mice by regulating miR-26a-5p. **A**, **B**, H&E staining (magnification: × 100) was performed to detect the lung injury degree in ALI mice (n = 8 per group). **C** Statistical analysis revealed the lung wet/dry weight ratio (n = 8 per group). **D**–**G** RT-qPCR and ELISA were applied to assess the levels of TNF-α and IL-1β in serum of mice (n = 8 per group) and in supernatant of PBECs. ^#^*P* < 0.05 compared with Sham + AAV-Mock + AAV-vector group, ^&^*P* < 0.05 compared with ALI + AAV-Mock + AAV-vector group, ^@^*P* < 0.05 compared with ALI + AAV-OIP5-AS1 + AAV-vector group in **B**–**E**; ^#^*P* < 0.05 compared with control + Mock + vector group, ^&^*P* < 0.05 compared with LPS + Mock + vector group, ^@^*P* < 0.05 compared with LPS + OIP5-AS1 + vector group in **F**, **G**

H&E staining results showed that miR-26a-5p upregulation alleviated the OIP5-AS1 overexpression caused lung injury in ALI mice (Fig. 6A, B). The lung wet/drying ratio was increased after OIP5-AS1 upregulation, while miR-26a-5p overexpression rescued the effect of OIP5-AS1 upregulation (Fig. 6C). Moreover, OIP5-AS1 upregulation increased the mRNA and protein levels of TNF-α and IL-1β, while miR-26a-5p overexpression significantly rescued the effects in ALI mice (Fig. 6D, E), and in LPS treated PBECs (Fig. 6F, G). Additional file 2: Fig. S4A, B revealed that OIP5-AS1 increased TNF-α and IL-1β expression by binding with miR-26a-5p in WI-38 and TC-1 cells.

### OIP5-AS1 facilitates cell apoptosis via binding with miR-26a-5p

**Fig. 7:**
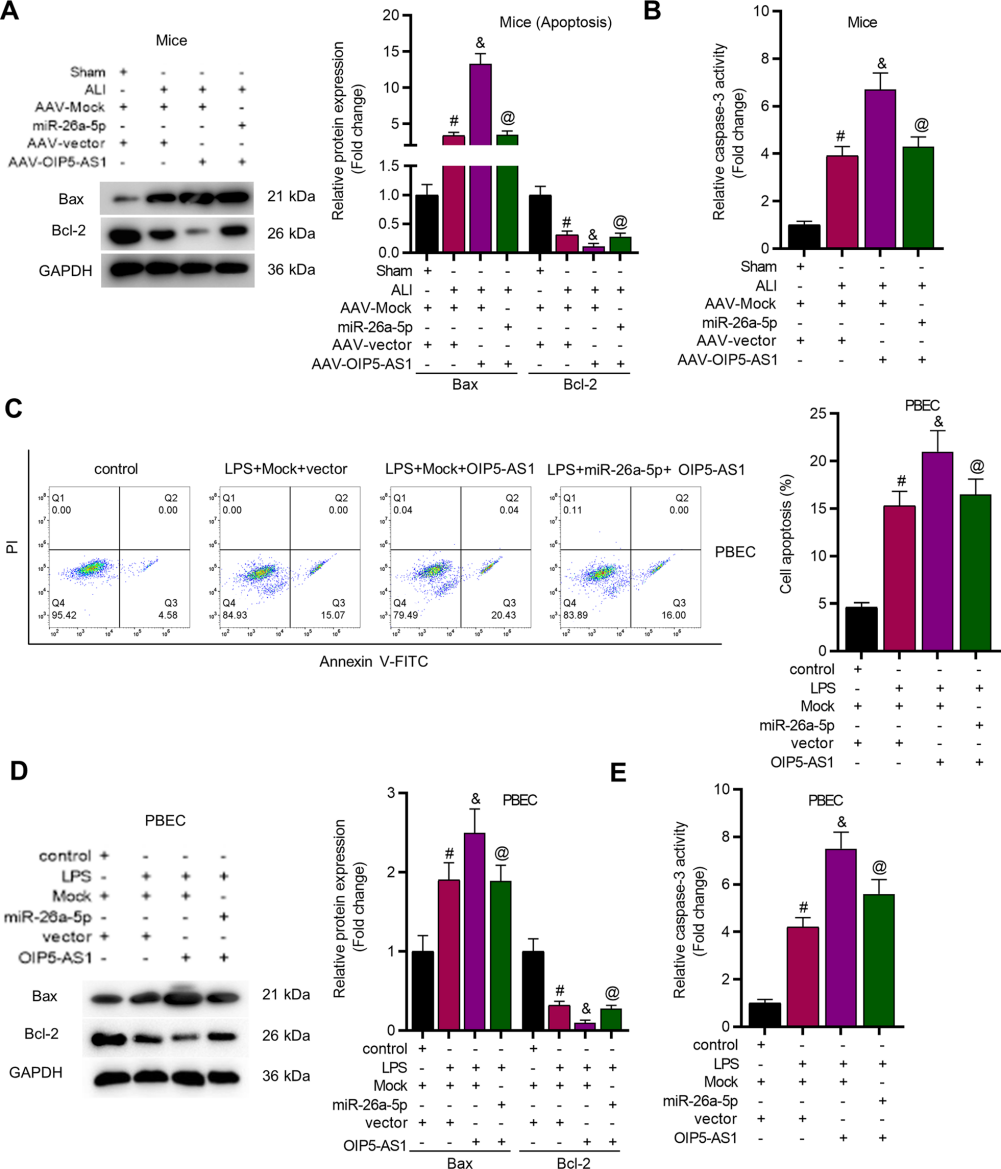
OIP5-AS1 accelerated cell apoptosis via regulating miR-26a-5p. **A** The protein expression of Bax and Bcl-2 in lung tissues of ALI mice (n = 8 per group) was assessed by western blot assay. **B** Relative activity of caspase-3 in lung tissues of ALI mice (n = 8 per group). **C** Flow cytometry assay was utilized to reveal cell apoptosis rate of PBECs. **D** The protein expression of apoptosis genes in PBECs was revealed by western blot assay. **E** The activity of caspase-3 in PBECs was detected. ^#^*P* < 0.05 compared with Sham + AAV-Mock + AAV-vector group, ^&^*P* < 0.05 compared with ALI + AAV-Mock + AAV-vector group, ^@^*P* < 0.05 compared with ALI + AAV-OIP5-AS1 + AAV-vector group in **A**, **B**; ^#^*P* < 0.05 compared with control + Mock + vector group, ^&^*P* < 0.05 compared with LPS + Mock + vector group, ^@^*P* < 0.05 compared with LPS + Mock + OIP5-AS1 group in **C**–**E**

Overexpression of miR-26a-5p reversed the effects of OIP5-AS1 upregulation on Bax and Bcl-2 protein levels in lung tissues of ALI mice (Fig. 7A). MiR-26a-5p overexpression rescued the promotive effect of OIP5-AS1 on caspase-3 activity in lung tissues of ALI mice (Fig. 7B). Additionally, miR-26a-5p upregulation counteracted the promotive influence of OIP5-AS1 on cell apoptosis in LPS-stimulated PBECs (Fig. 7C). The level of Bax was increased while the level of Bcl-2 was suppressed by OIP5-AS1 overexpression in PBECs, and the result was recovered by the upregulation of miR-26a-5p (Fig. 7D). As presented in Fig. 7E, the caspase-3 activity was increased after LPS treatment in PBECs, further increased by OIP5-AS1 upregulation, and repressed by miR-26a-5p mimics. Additional file 2: Fig. S4C–F revealed that OIP5-AS1 increased apoptosis of WI-38 and TC-1 cells by binding with miR-26a-5p.

## Discussion

ALI is a severe illness that threats health and lives worldwide because of the high incidence and mortality [40]. TLR4, also known as TOLL [41], is a member of the Toll-like receptor family that plays a fundamental role in pathogen recognition and activation of innate immunity [42, 43]. Moreover, the toll-like receptors exhibit different patterns of expression and involve in LPS-induced signal transduction events in most gram-negative bacteria [44]. Previous studies have revealed that TLR4 displayed high level in ALI tissues and significantly facilitated the inflammatory response and cell apoptosis in ALI [45, 46]. Similarly, the upregulation of TLR4 was also identified in ALI mice and LPS-stimulated PBECs in our research. An excessive accumulation and retention of neutrophils in inflamed tissue can cause severe tissue injuries in the later stages of inflammation [47]. Resident alveolar macrophages also play a significant role in ALI. In the acute phase of ALI, resident alveolar macrophages that typically express the alternatively activated phenotype, shift into the classically activated phenotype and release various potent proinflammatory mediators [48]. The present study revealed that TLR4 expression was higher in macrophages and neutrophils isolated from the lungs in the ALI mouse model than sham-operated mice.

We then further explored the upstream regulatory mechanism of TLR4. Considering TLR4 was reported to be inhibited by multiple miRNAs including miR-214 and miR-1178 [49, 50], we then searched the potential miRNAs that can bind with TLR4, and miR-26a-5p was confirmed to directly target TLR4 in PBECs. Moreover, miR-26a-5p negatively regulated TLR4 expression. More importantly, miR-26a-5p overexpression inhibited the effects of TLR4 on inflammatory response and cell apoptosis of LPS-stimulated PBECs. In summary, miR-26a-5p can regulate the development of ALI via targeting TLR4.

It is widely accepted that lncRNAs are able to act as miRNA “sponges” to competitively combine with miRNAs to release target mRNAs [51]. In ALI, taurine up-regulated 1 was identified to alleviate sepsis-induced inflammation and apoptosis via the miR-34b-5p/ growth factor receptor bound protein 2-associated protein 1 axis [52]. In addition, downregulated cancer susceptibility 9 promotes cell apoptosis by the miR-195-5p/pyruvate dehydrogenase kinase 4 axis [53]. In our study, we confirmed that OIP5-AS1 can bind with miR-26a-5p using RIP and luciferase reporter assays. Previously, OIP5-AS1 was reported to exert different effects on cell injury and inflammatory response in different pathogenesis. In rheumatoid arthritis, OIP5-AS1 was proposed to inhibit inflammatory response by suppressing the toll like receptor 3-nuclear factor kappa B pathway [23]. In atherosclerosis, OIP5-AS1 was confirmed to facilitate cell apoptosis and inflammation by activating the nuclear factor kappa B pathway [24]. In the current study, we identified that OIP5-AS1 negatively regulated the expression of miR-26a-5p. Additionally, OIP5-AS1 upregulation increased the lung injury scores, the lung wet/dry weight ratio, productions of proinflammatory factors and cell apoptosis in ALI, while miR-26a-5p elevation counteracted these effects.

In summary, our results revealed that OIP5-AS1 aggravated ALI by promoting inflammation and apoptosis by regulating the miR-26a-5p/TLR4 axis, which offers new insights into the therapeutic strategy of ALI.

## Supplementary Information

**Additional file 1.** Uncropped and unprocessed western blots.

**Additional file 2.** The role of the OIP5-AS1/miR-26a-5p/TLR4 axis in inflammatory response and apoptosis of WI-38 and TC-1 cells.

## Data Availability

The datasets used and/or analyzed during the current study available from the corresponding author on reasonable request.
