# Supplementary material for: Long non-coding RNA OIP5-AS1 aggravates acute lung injury by promoting inflammation and cell apoptosis via regulating the miR-26a-5p/TLR4 axis
Source: BMC Pulm Med. 2021 Jul 14;21:236. doi: 10.1186/s12890-021-01589-1 (PMC8281572; doi:10.1186/s12890-021-01589-1)

1C

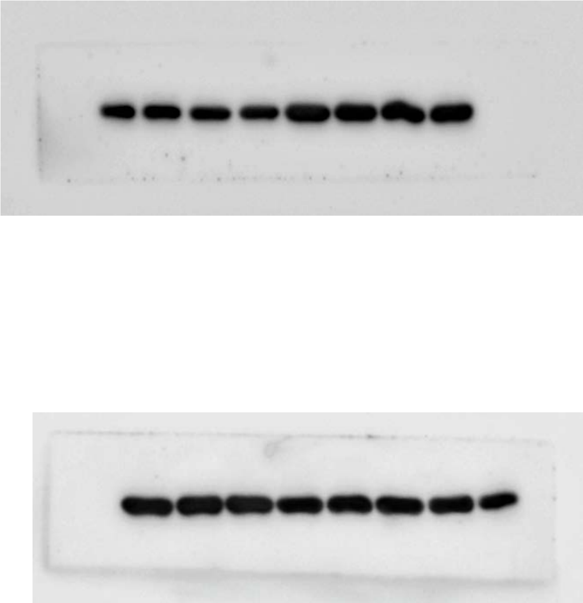

1F

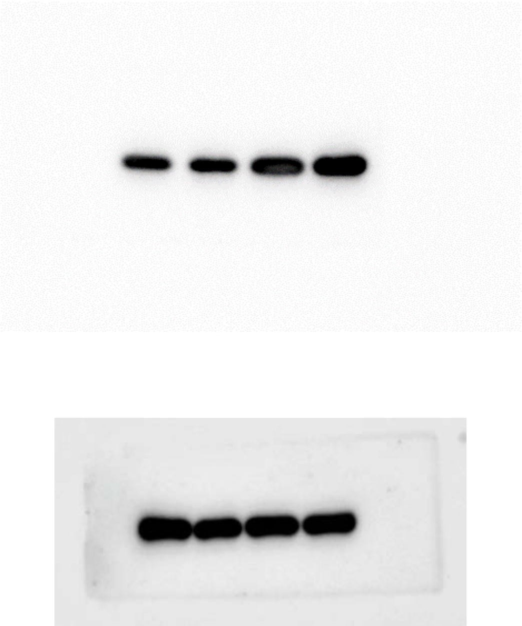

2E

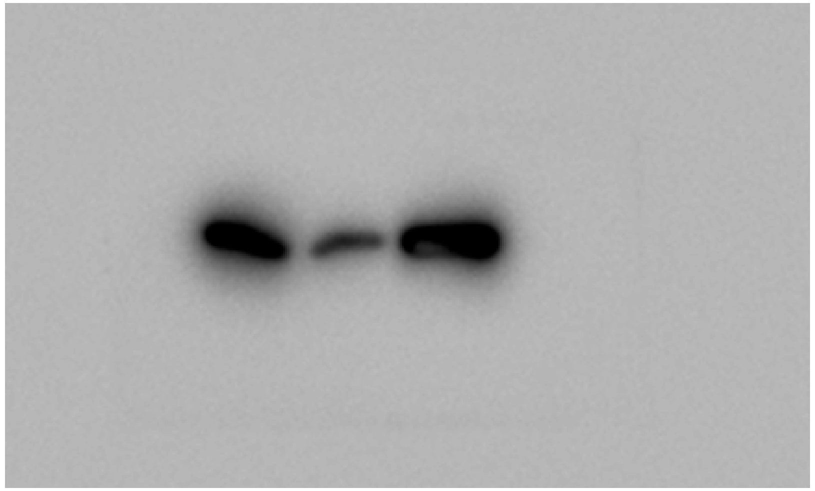

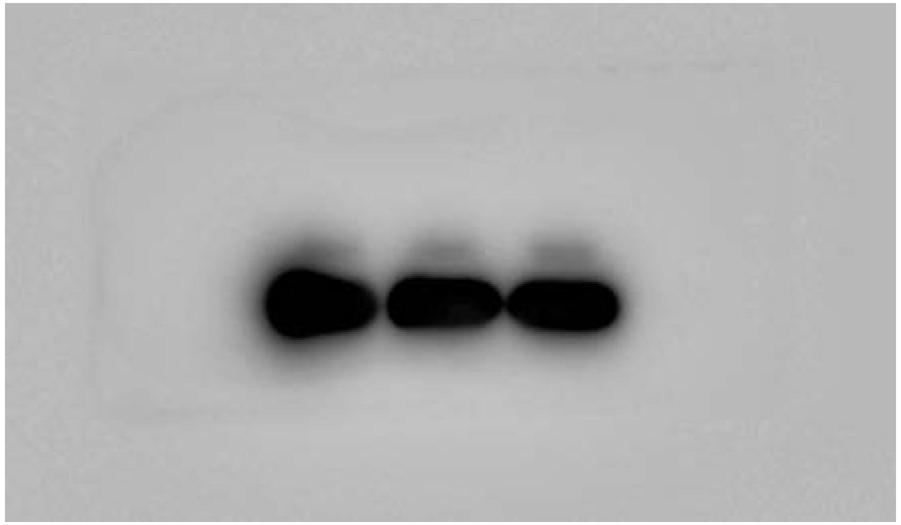

7A

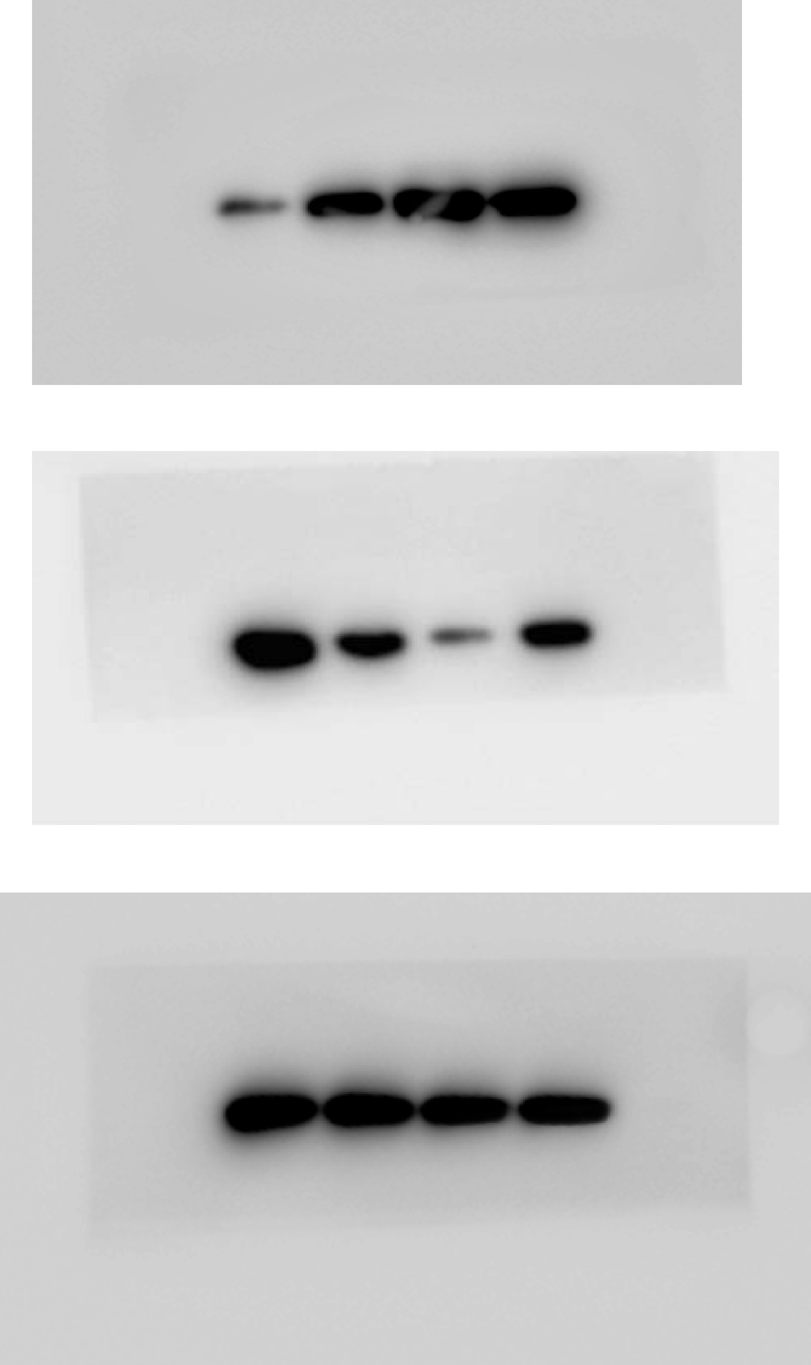

7D

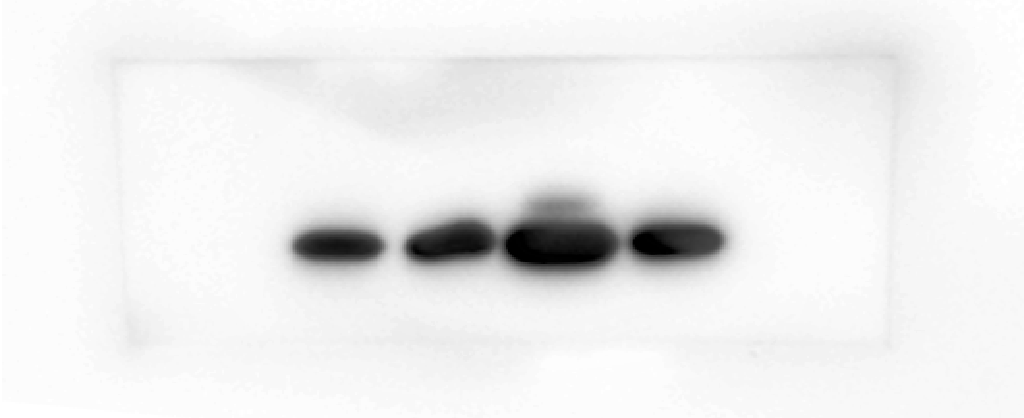

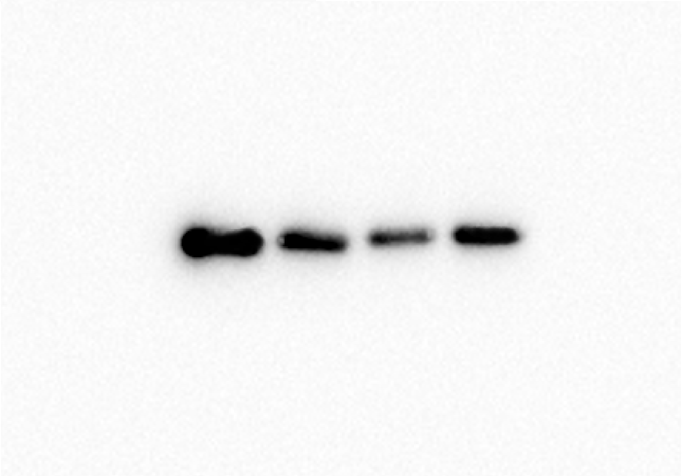

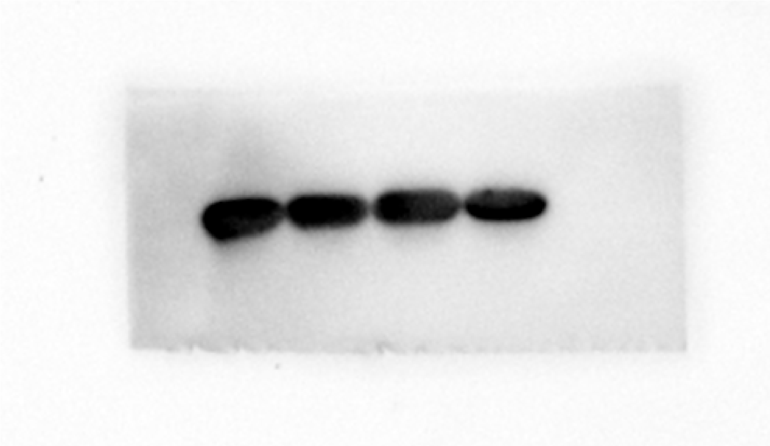

Supplementary Figure 1A

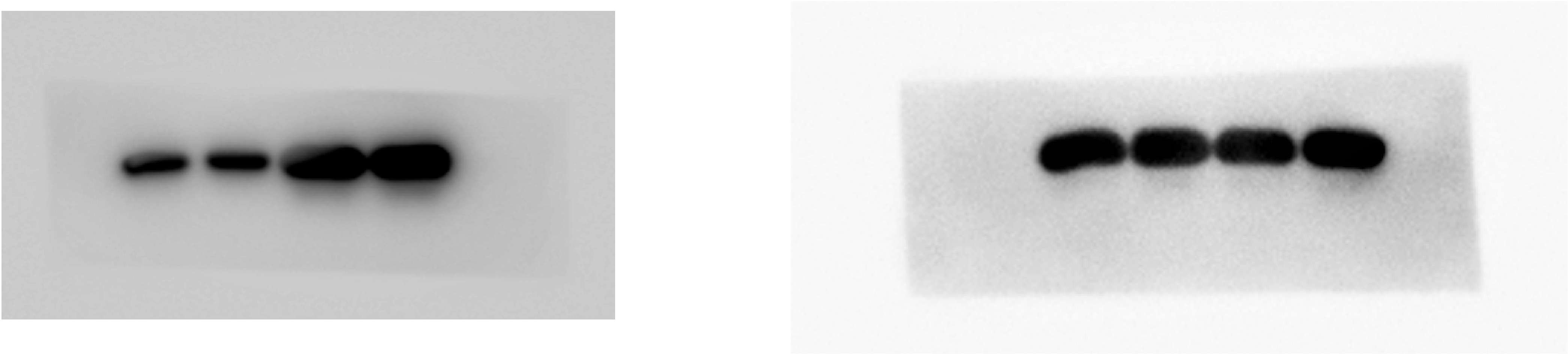

Supplementary Figure 1E

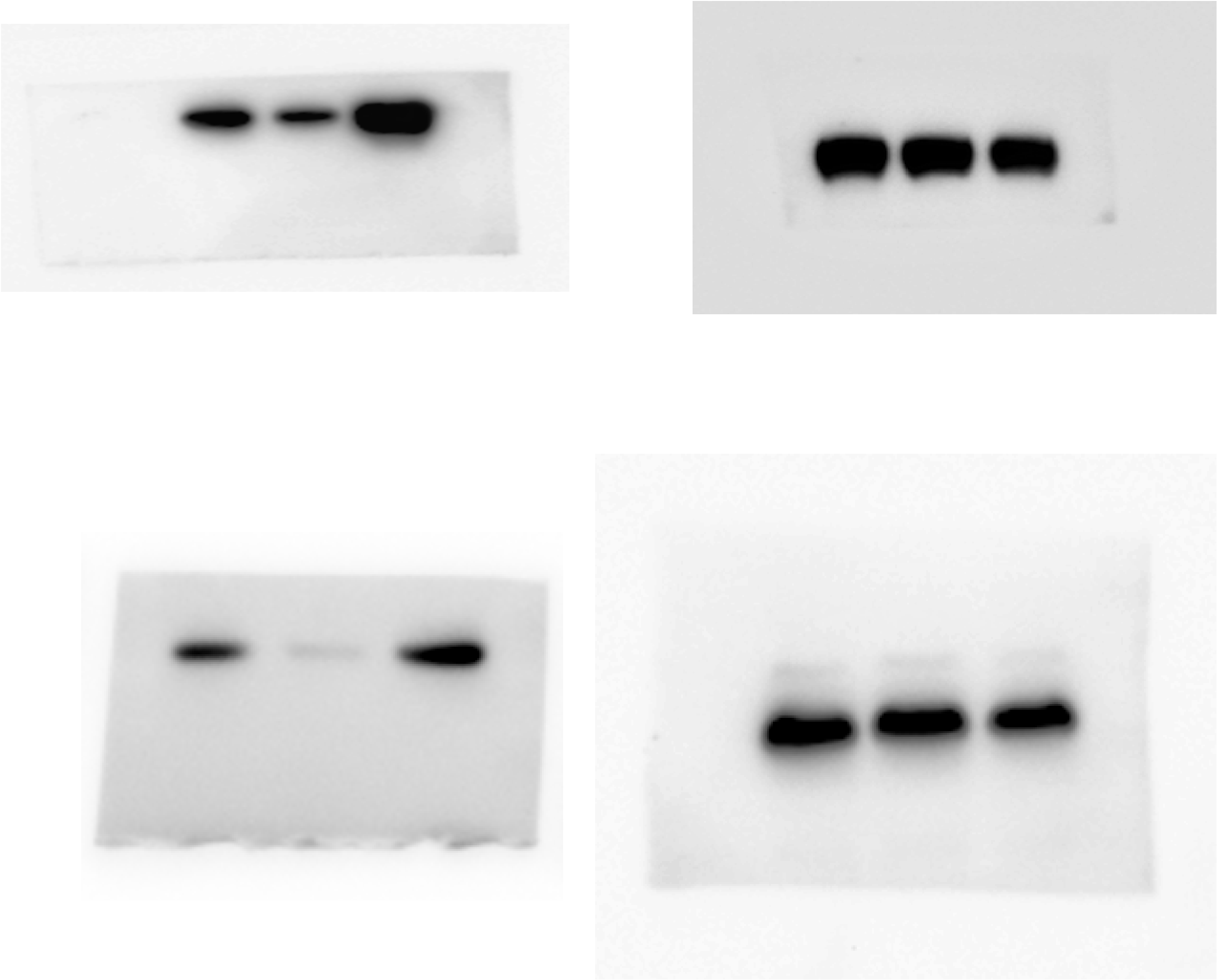

Supplementary Figure 4D

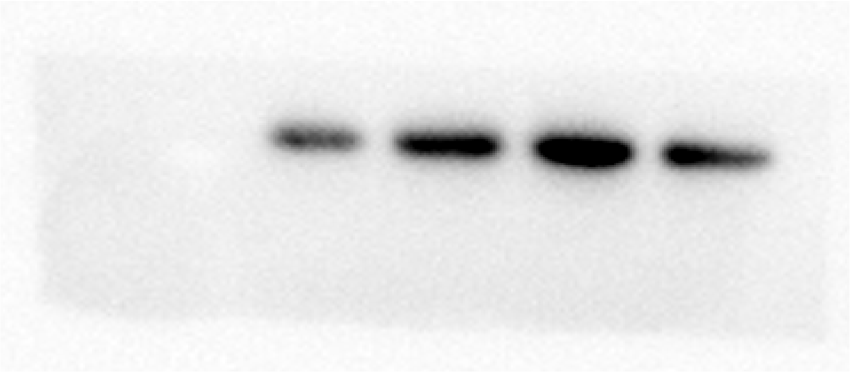

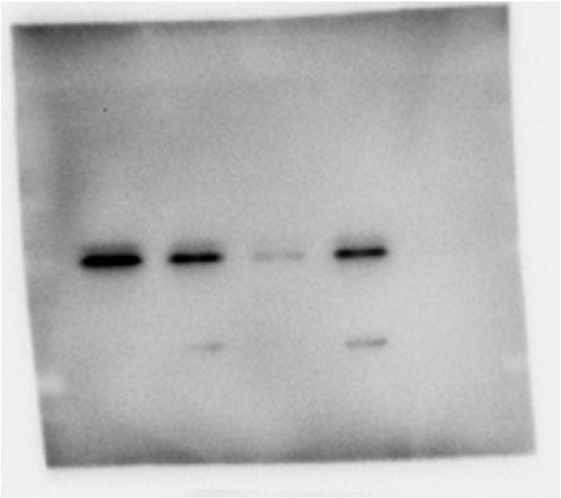

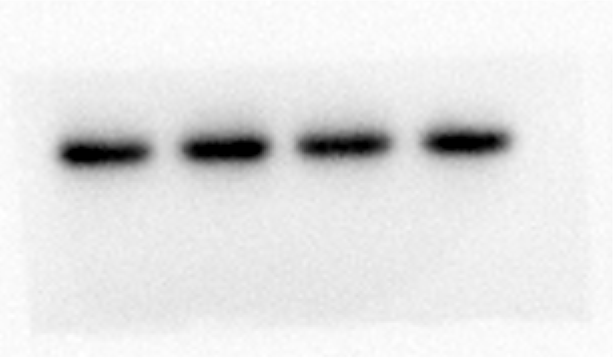

Supplementary Figure 4E

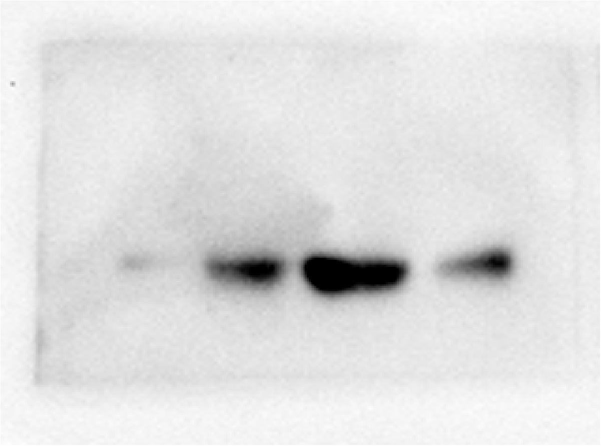

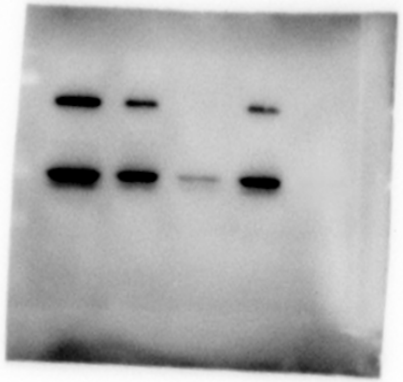

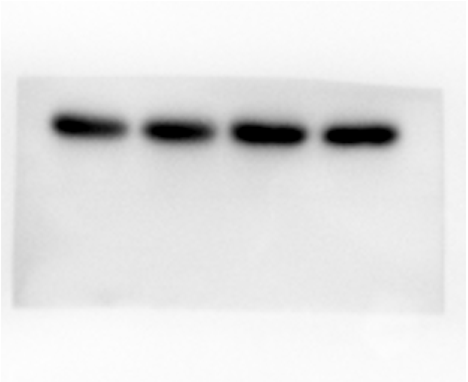

Supplement: Supplementary file 1 — Additional file 1. Uncropped and unprocessed western blots. [file 12890_2021_1589_MOESM1_ESM.pdf]
