# Supplementary material for: Long non-coding RNA OIP5-AS1 aggravates acute lung injury by promoting inflammation and cell apoptosis via regulating the miR-26a-5p/TLR4 axis
Source: BMC Pulm Med. 2021 Jul 14;21:236. doi: 10.1186/s12890-021-01589-1 (PMC8281572; doi:10.1186/s12890-021-01589-1)
Supplement: Supplementary file 2 — Additional file 2. The role of the OIP5-AS1/miR-26a-5p/TLR4 axis in inflammatory response and apoptosis of WI-38 and TC-1 cells. [file 12890_2021_1589_MOESM2_ESM.docx]

**
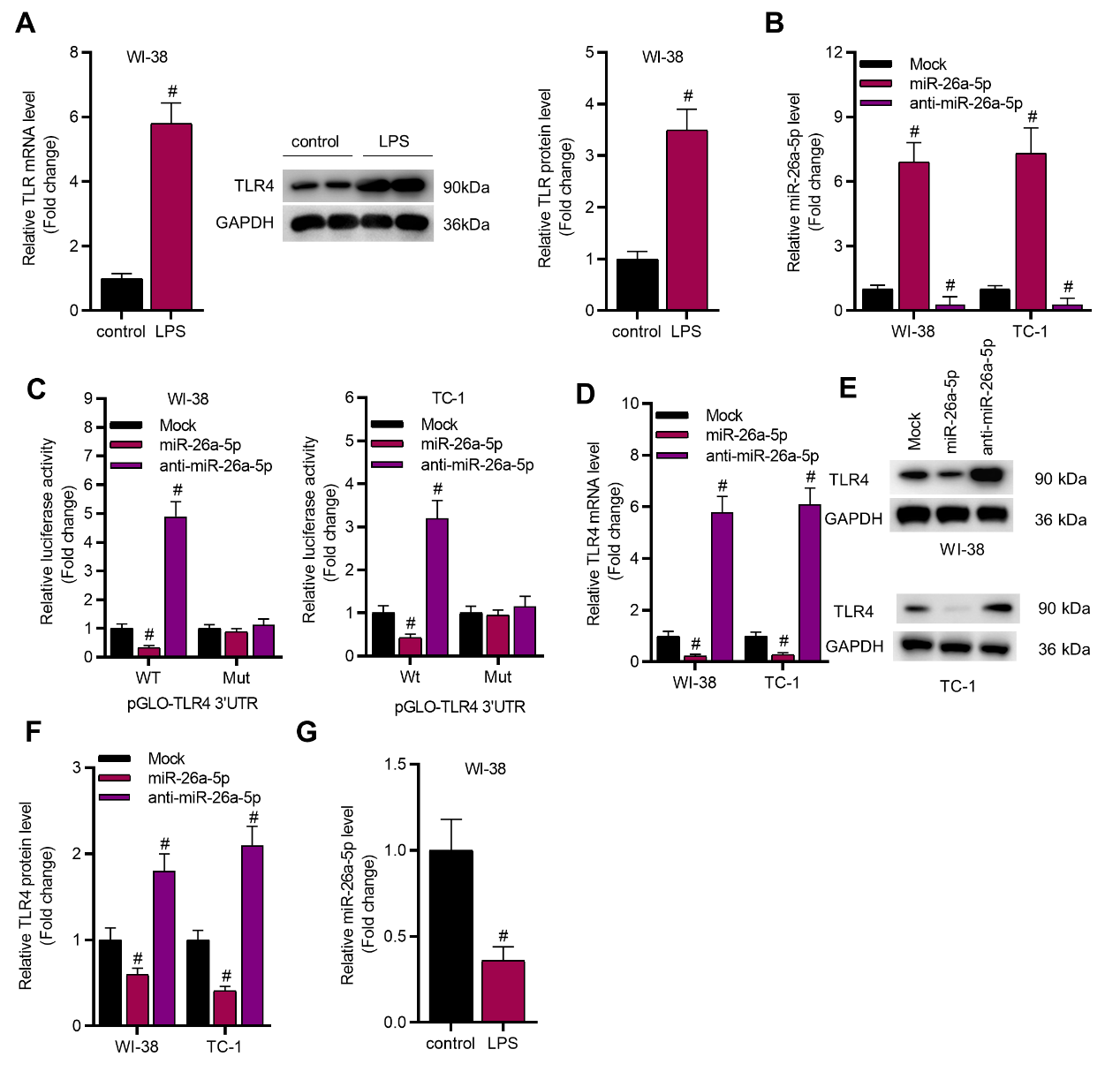
**

**Supplementary Figure 1 TLR4 was negatively modulated by miR-26a-5p in WI-38 and TC-1 cells.** A, The mRNA and protein expression of TLR4 in LPS-stimulated WI-38 cells was detected by RT-qPCR and western blot analyses. B, RT-qPCR was conducted to assess the efficiency of miR-26a-5p overexpression and miR-26a-5p knockdown in WI-38 and TC-1 cells. C, Luciferase reporter assay was performed to verify the interaction between miR-26a-5p and TLR4 in WI-38 and TC-1 cells. D-F, RT-qPCR and western blot assay were applied to detect the effects of miR-26a-5p overexpression and miR-26a-5p knockdown on the mRNA and protein expression of TLR4 in WI-38 and TC-1 cells. G, The expression of miR-26a-5p in LPS-treated WI-38 cells was assessed by RT-qPCR analysis. ^#^P< 0.05 compared with control group in Figure A, G and with Mock group in Figures B, C, D, F.

**
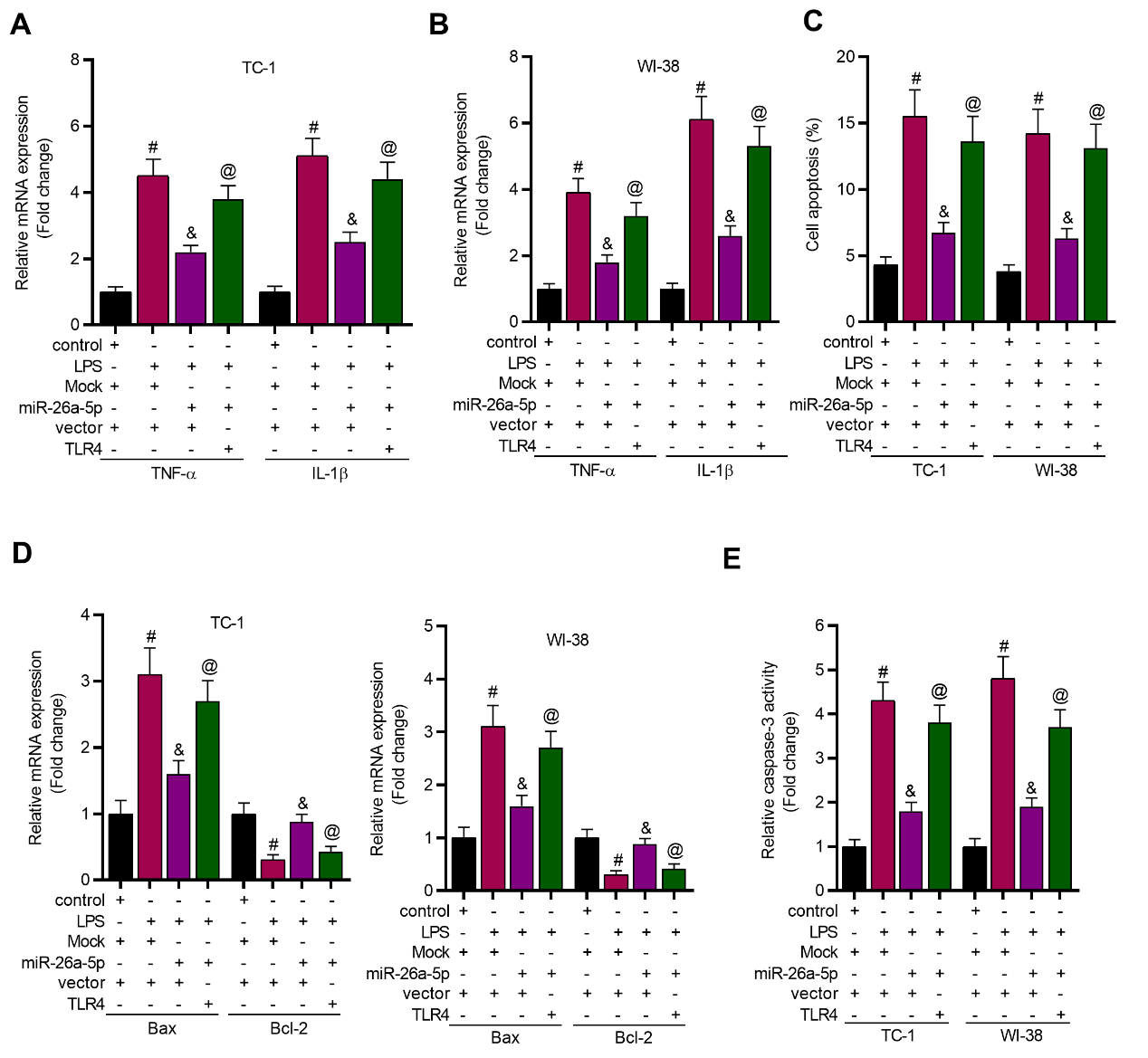
**

**Supplementary Figure 2 MiR-26a-5p regulated inflammatory response and apoptosis of WI-38 and TC-1 cells by modulating TLR4.** A-B, RT-qPCR was performed to evaluate the levels of TNF-α and IL-1β in WI-38 and TC-1 cells. C, Flow cytometry assay was utilized to reveal apoptosis rate of WI-38 and TC-1 cells. D, Bax and Bcl-2 expression in WI-38 and TC-1 cells was calculated by RT-qPCR. E, The activity of caspase-3 in WI-38 and TC-1 cells was detected. ^#^P< 0.05 compared with control + Mock + vector group, ^&^P< 0.05 compared with LPS + Mock + vector group, ^@^ P< 0.05 compared with LPS + miR-26a-5p + vector group.

**
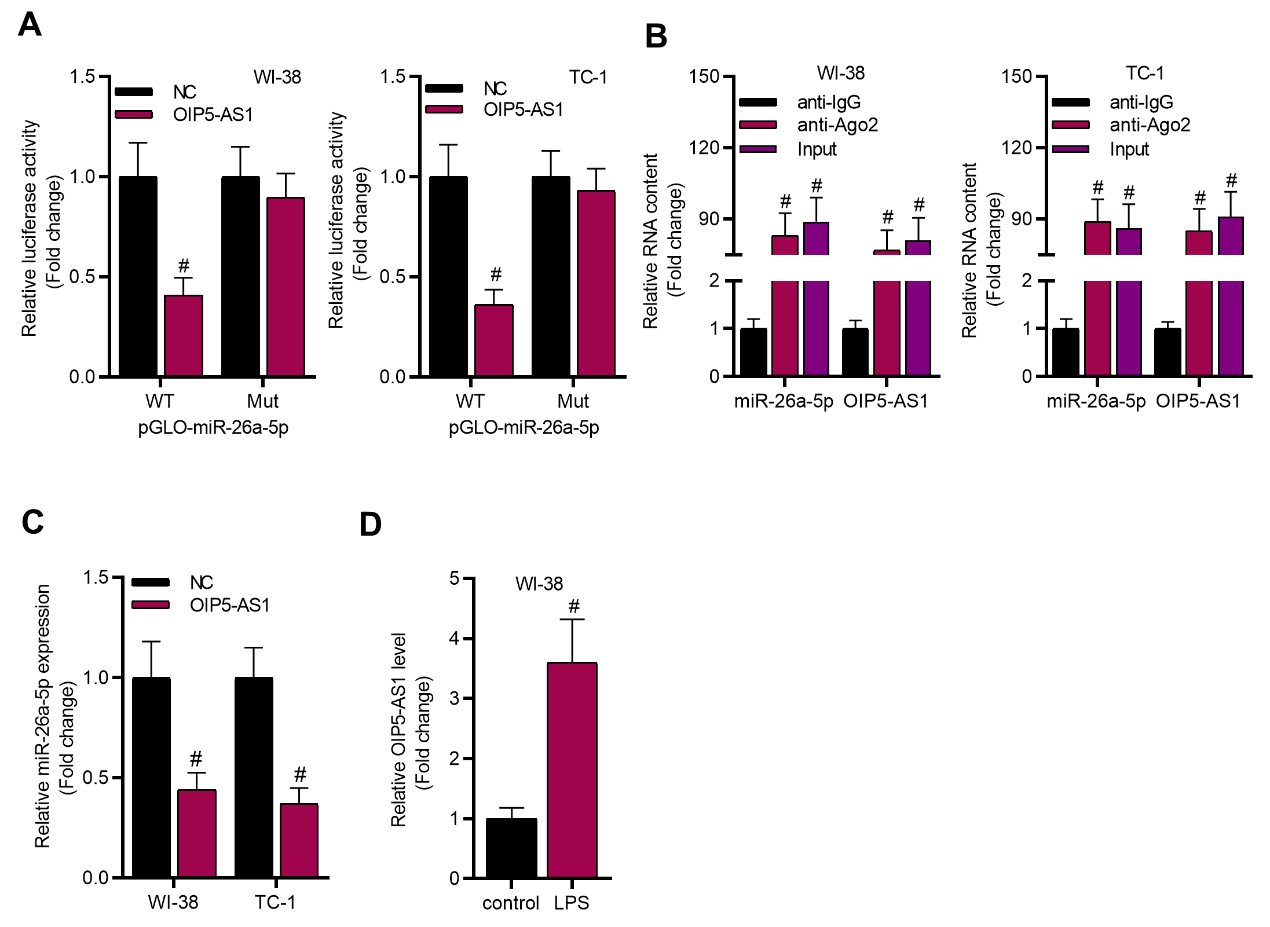
**

**Supplementary Figure 3 OIP5-AS1 bound with miR-26a-5p in WI-38 and TC-1 cells.** A-B, The interaction between miR-26a-5p and OIP5-AS1 in WI-38 and TC-1 cells was demonstrated by luciferase reporter and RIP assays. ^#^P< 0.05 compared with NC group or anti-IgG group. C, The effects of overexpressed OIP5-AS1 on miR-26a-5p expression in WI-38 and TC-1 cells were estimated by RT-qPCR. ^#^P< 0.05 compared with NC group. D, RT-qPCR analysis was conducted to evaluate the level of OIP5-AS1 in LPS-stimulated WI-38 cells. ^#^P< 0.05 compared with control group.

**
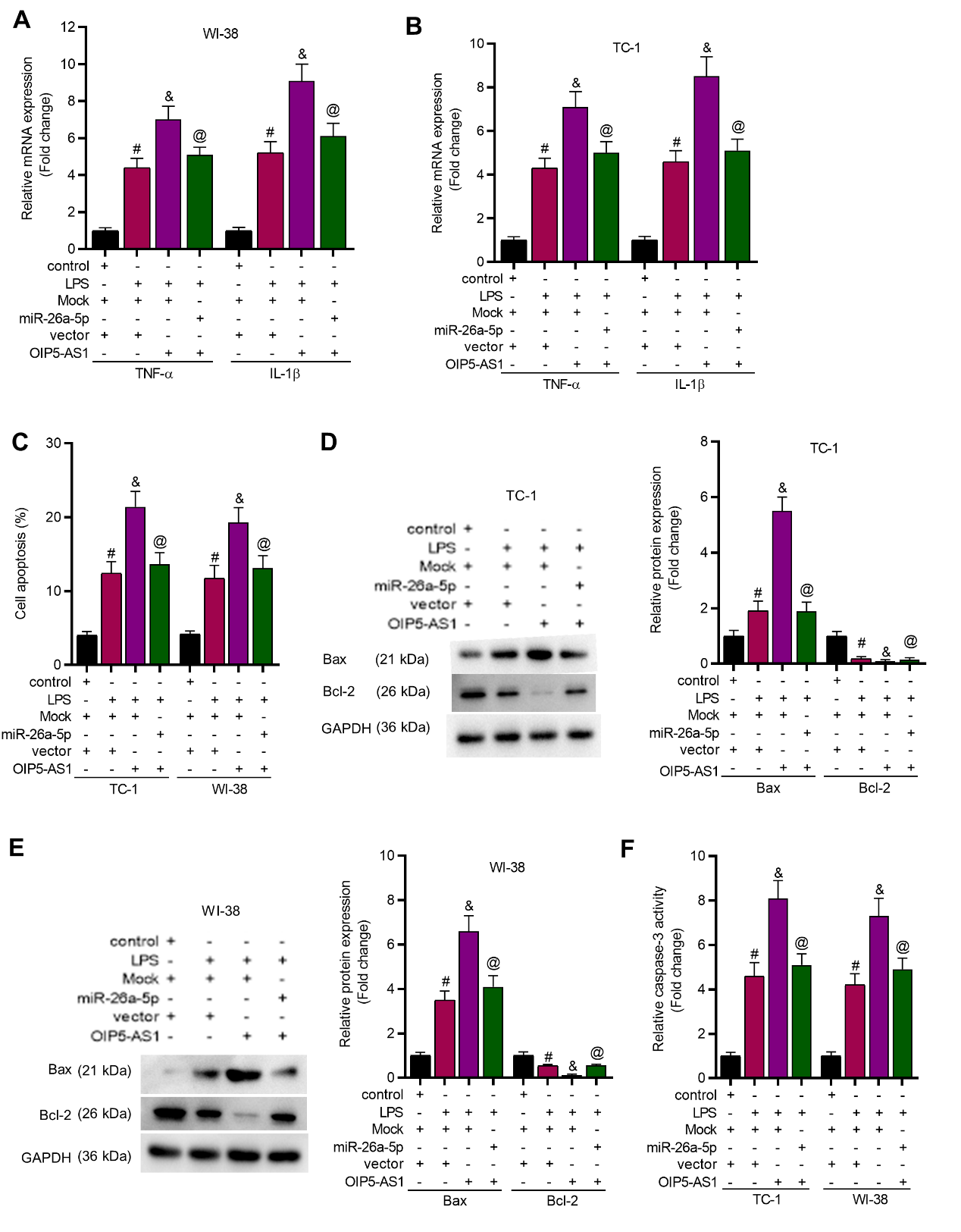
**

**Supplementary Figure 4 OIP5-AS1 regulated inflammatory response and apoptosis of WI-38 and TC-1 cells by modulating miR-26a-5p.** A-B, RT-qPCR was performed to evaluate the levels of TNF-α and IL-1β in WI-38 and TC-1 cells. C, Flow cytometry assay was utilized to reveal apoptosis rate of WI-38 and TC-1 cells. D-E, Bax and Bcl-2 expression in WI-38 and TC-1 cells was calculated by RT-qPCR. F, The activity of caspase-3 in WI-38 and TC-1 cells was detected. ^#^P< 0.05 compared with control + Mock + vector group, ^&^P< 0.05 compared with LPS + Mock + vector group, ^@^ P< 0.05 compared with LPS + Mock + OIP5-AS1 group.

**Supplementary table 1 Potential lncRNAs binding with miR-26a-5p predicted from starBase**

| **miRNAid** | **miRNAname** | **geneID** | **geneName** | **geneType** |
| --- | --- | --- | --- | --- |
| MIMAT0000082 | hsa-miR-26a-5p | ENSG00000272084 | AL137127.1 | 3prime_overlapping_ncRNA |
| MIMAT0000082 | hsa-miR-26a-5p | ENSG00000273026 | AL358472.3 | antisense |
| MIMAT0000082 | hsa-miR-26a-5p | ENSG00000234741 | GAS5 | processed_transcript |
| MIMAT0000082 | hsa-miR-26a-5p | ENSG00000206573 | THUMPD3-AS1 | antisense |
| MIMAT0000082 | hsa-miR-26a-5p | ENSG00000245060 | LINC00847 | lincRNA |
| MIMAT0000082 | hsa-miR-26a-5p | ENSG00000228223 | HCG11 | lincRNA |
| MIMAT0000082 | hsa-miR-26a-5p | ENSG00000224843 | LINC00240 | lincRNA |
| MIMAT0000082 | hsa-miR-26a-5p | ENSG00000203875 | SNHG5 | processed_transcript |
| MIMAT0000082 | hsa-miR-26a-5p | ENSG00000226816 | AC005082.1 | lincRNA |
| MIMAT0000082 | hsa-miR-26a-5p | ENSG00000243107 | AC000120.1 | sense_overlapping |
| MIMAT0000082 | hsa-miR-26a-5p | ENSG00000245910 | SNHG6 | processed_transcript |
| MIMAT0000082 | hsa-miR-26a-5p | ENSG00000269821 | KCNQ1OT1 | antisense |
| MIMAT0000082 | hsa-miR-26a-5p | ENSG00000245532 | NEAT1 | lincRNA |
| MIMAT0000082 | hsa-miR-26a-5p | ENSG00000251562 | MALAT1 | lincRNA |
| MIMAT0000082 | hsa-miR-26a-5p | ENSG00000251562 | MALAT1 | lincRNA |
| MIMAT0000082 | hsa-miR-26a-5p | ENSG00000279742 | AP000974.1 | TEC |
| MIMAT0000082 | hsa-miR-26a-5p | ENSG00000258210 | AC144548.1 | processed_transcript |
| MIMAT0000082 | hsa-miR-26a-5p | ENSG00000235423 | AC068768.1 | antisense |
| MIMAT0000082 | hsa-miR-26a-5p | ENSG00000258824 | AL122035.1 | antisense |
| MIMAT0000082 | hsa-miR-26a-5p | ENSG00000247556 | OIP5-AS1 | processed_transcript |
| MIMAT0000082 | hsa-miR-26a-5p | ENSG00000103472 | RRN3P2 | processed_transcript |
| MIMAT0000082 | hsa-miR-26a-5p | ENSG00000264772 | AC016876.2 | processed_transcript |
| MIMAT0000082 | hsa-miR-26a-5p | ENSG00000215769 | ARHGAP27P1-BPTFP1-KPNA2P3 | processed_transcript |
| MIMAT0000082 | hsa-miR-26a-5p | ENSG00000268205 | AC005261.1 | lincRNA |
| MIMAT0000082 | hsa-miR-26a-5p | ENSG00000260032 | NORAD | lincRNA |
| MIMAT0000082 | hsa-miR-26a-5p | ENSG00000223768 | LINC00205 | bidirectional_promoter_lncRNA |
| MIMAT0000082 | hsa-miR-26a-5p | ENSG00000093100 | AC016026.1 | processed_transcript |
| MIMAT0000082 | hsa-miR-26a-5p | ENSG00000253352 | TUG1 | bidirectional_promoter_lncRNA |
| MIMAT0000082 | hsa-miR-26a-5p | ENSG00000253352 | TUG1 | bidirectional_promoter_lncRNA |
| MIMAT0000082 | hsa-miR-26a-5p | ENSG00000261409 | AL035425.3 | sense_overlapping |
